# Using the AR-V7 biomarker to determine treatment in metastatic castrate resistant prostate cancer, a feasibility randomised control trial, conclusions from the VARIANT trial

**DOI:** 10.3310/nihropenres.13284.2

**Published:** 2023-01-10

**Authors:** Paul Gravestock, Emma Clark, Miranda Morton, Shirya Sharma, Holly Fisher, Jenn Walker, Ruth Wood, Helen Hancock, Nichola Waugh, Aislinn Cooper, Rebecca Maier, John Marshall, Robert Chandler, Amit Bahl, Simon Crabb, Suneil Jain, Ian Pedley, Rob Jones, John Staffurth, Rakesh Heer

**Affiliations:** 1 Newcastle upon Tyne Hospitals NHS Foundation Trust, Newcastle upon Tyne, Tyne and Wear, NE3 3HD, UK; 2 Translational and Clinical Research Institute, NU Cancer, Newcastle upon Tyne, Tyne and Wear, NE1 7RU, UK; 3 Newcastle Clinical Trials Unit, Newcastle University, Newcastle upon Tyne, Tyne and Wear, NE2 4AE, UK; 4 Population Health Sciences, Newcastle University, Newcastle upon Tyne, Tyne and Wear, NE1 7RU, UK; 5 Velindre University NHS Trust, Cardiff, CF15 7QZ, UK; 6 Trial Management Group, VARIANT Trial, Newcastle upon Tyne, Tyne and Wear, NE1 7RU, UK; 7 University Hospitals Bristol NHS Foundation Trust, Bristol, BS1 3NU, UK; 8 University of Southampton, Southampton, Hampshire, SO17 1BJ, UK; 9 Queens University Belfast, Belfast, BT7 1NN, UK; 10 Institute of Cancer Services, University of Glasgow, Glasgow, G12 0YN, UK; 11 Division of Cancer and Genetics, Cardiff University, Cardiff, CF14 4XN, UK

**Keywords:** prostatic neoplasms, castration-resistant, biomarkers, feasibility studies, male

## Abstract

**Background:**

Prostate cancer is the most commonly diagnosed malignancy in the UK. Castrate resistant prostate cancer (CRPC) can be difficult to manage with response to next generation hormonal treatment variable. AR-V7 is a protein biomarker that can be used to predict response to treatment and potentially better inform management in these patients. Our aim was to establish the feasibility of conducting a definitive randomised controlled trial comparing the clinical utility of AR-V7 biomarker assay in personalising treatments for patients with metastatic CRPC within the United Kingdom (UK) National Health Service (NHS). Due to a number of issues the trial was not completed successfully, we aim to discuss and share lessons learned herein.

**Methods:**

We conducted a randomised, open, feasibility trial, which aimed to recruit 70 adult men with metastatic CRPC within three secondary care NHS trusts in the UK to be run over an 18-month period. Participants were randomised to personalised treatment based on AR-V7 status (intervention) or standard care (control). The primary outcome was feasibility, which included: recruitment rate, retention and compliance. Additionally, a baseline prevalence of AR-V7 expression was to be estimated.

**Results:**

Fourteen participants were screened and 12 randomised with six into each arm over a nine-month period. Reliability issues with the AR-V7 assay meant prevalence was not estimated. Due to limited recruitment the study did not complete to target.

**Conclusions:**

Whilst the trial did not complete to target, we have ascertained that men with advanced cancer are willing to take part in trials utilising biomarker guided treatment. A number of issues were identified that serve as important learning points in future clinical trials.

## Plain English summary

In advanced prostate cancer patients are commonly treated with hormone therapy to control the cancer growth. Eventually this treatment stops working, and the next steps involve treatment with either more advanced hormone therapy (abiraterone and enzalutamide) or with chemotherapy/radiotherapy.

The VARIANT trial used a blood test to check for a marker in the blood called the AR-V7 protein which may help predict which treatment option is better in these patients. By doing this trial we wanted to explore if patients and their doctors were willing to use this test to help decide the best treatment. We planned to recruit 70 men for the study.

Patients who agreed to take part were put into one of two groups: (1) treatment guided by the AR-V7 test or (2) treatment as usual, decided by both doctor and patient. Blood samples were collected when the patient agreed to take part in the trial (after signing their consent), at 12 weeks and at 24 weeks after they started treatment. The blood samples were sent to the Newcastle University labs to test for AR-V7 positivity. Extra blood samples that were not used were stored in a biobank to be used in future prostate cancer research. Participants were asked to complete questionnaires throughout the trial. The trial took place across three NHS organisations across the UK, in Newcastle, Cardiff and Glasgow.

The trial was planned to start recruiting participants at all three sites in October 2018. Unfortunately, due to unforeseen delays recruitment did not start until July 2019 and all three sites did not open until February 2020. A total of 12 patients were recruited.

The trial showed patients were willing to be randomised to the trial to allow their treatment to be guided by a blood test. Unfortunately, due to a number of delays and difficulties with recruitment and a change in the standard treatment the study did not fully meet its outcomes.

## Introduction

Prostate cancer is the most diagnosed malignancy in the United Kingdom (UK) and the second most common cause of cancer mortality ^1^. Whilst overall survival rates are high, metastatic prostate cancer is incurable with poor five-year survival rates ^2^. Treatment for metastatic prostate cancer includes medical or surgical castration, the former consists of androgen deprivation therapy (ADT) which aims to block production of testosterone and/or block its action on testosterone receptors. In prostatic tissue, testosterone acts on cells to promote growth and proliferation, blocking these signals with ADT leads androgen sensitive cells to undergo apoptosis ^3^. When metastatic prostate cancer responds to ADT it is termed metastatic hormone sensitive prostate cancer (mHSPC). Whilst a good response to ADT is often seen initially, it is inevitable that the disease begins to progress despite treatment to become what is then termed metastatic castrate resistant prostate cancer (mCRPC) ^3, 4^.

When VARIANT was conceived the standard of care for mHSPC, for most patients, was ADT alone, at the subsequent development of castrate resistance additional treatment would then consist of either next generation androgen receptor targeted agents (ARTAs), non-hormonal treatment with chemotherapy or drug delivered radiotherapy (radium 233) ^5^. ARTAs such as abiraterone or enzalutamide are typically the preferred option as they generally have less side effects. However, response is variable with a proportion of patients resistant to the treatment primarily and all patients eventually becoming treatment refractory ^3^. Predicting a positive response in individual patients is challenging and failed response, disease progression and uncertainty around treatment can be difficult for patients.

One method to better determine effective therapy for these patients has been proposed in the form of monitoring levels of androgen receptor splice variant 7 (AR-V7). Androgen receptor splice variants are variations of the androgen receptor protein which lack a portion of the normal ligand binding domain and allow signalling despite lack of activation by a binding ligand ^6– 9^. AR-V7 is an example of one these variants and has been found to have a higher expression in prostate tissue of patients with mCRPC than in those who are hormone naïve and has a strong association with hormone resistance and metastatic disease ^9, 10^. Moreover AR-V7, which can be detected on circulating tumour cells (CTCs) within patients’ blood samples, has been implicated in resistance to abiraterone and enzalutamide ^11, 12^.

The VARIANT randomised controlled trial (RCT) was designed to assess the feasibility of utilising the AR-V7 biomarker in order to determine the optimal treatment pathway, treating with ARTAs in those patients likely to benefit and alternative treatment options to optimise disease control in patients in whom further hormonal treatments are likely to be futile. The primary objective was to establish feasibility in conducting a definitive randomised trial comparing AR-V7 biomarker-driven management with the current standard care in patients with mCRPC. The secondary objectives were to (1) estimate AR-V7 biomarker prevalence in the trial population to inform sample size calculations for a definitive randomised control trial; (2) assess recruitment, compliance and retention rates; (3) confirm outcome measures for a future definitive trial and establish trial data response rates, variability, and data quality; and (4) establish a blood biobank to include baseline, 12 and 24-week blood samples for future translational studies.

We aim to report these results and also discuss the reasons why the trial was not successfully completed to target with a view to share lessons learnt from our feasibility study.

## Methods

We conducted a randomised, open, feasibility trial, with participants recruited from three secondary care National Health Service (NHS) organisations in the UK: Velindre University NHS Trust, The Newcastle upon Tyne Hospitals NHS Foundation Trust and NHS Greater Glasgow and Clyde. This was registered with the ISRCTN trial registry on 12/08/2019, with the identifier: ISRCTN10246848 available at https://doi.org/10.1186/ISRCTN10246848. Favourable ethical opinion was obtained from the Wales National Research Ethics Service (NRES) Committee, reference: 18/WA/0419.

Patients were identified from urology/oncology clinical services and were approached about the trial during their routine clinic appointments. To be eligible for the study, patients were aged ≥18 years old with mCRPC and high-risk features clinically suitable for ARTA or chemotherapy. The eligibility criteria is published in full in the trial protocol which is available as open access ( https://pubmed.ncbi.nlm.nih.gov/31857319/) ^13^. The criteria includes: disease progression despite medical or surgical castration, suitability for treatment with at least one ARTA and one non-hormonal therapy and at least two high risk features. High risk features were defined as: age <60 years at time of diagnosis of metastatic disease, bone metastases present at time of diagnosis, Gleason score 8–10, presence of visceral metastases, PSA doubling time of less than 3 months, elevated alkaline phosphatase, Eastern Cooperative Oncology Group (ECOG) Performance status worse than or equal to 1, previous treatment for CRPC with docetaxal chemotherapy or ARTA ^13^.

The trial was designed as a feasibility trial according to the definition of Eldridge *et al*. (2016) ^14^. Feasibility includes the deliverability of the intervention and in this case, assessment of the frequency of the positive assay measurements (predicted at approximately 30%). The target sample size was designed according to external pilot RCT recommendation by Teare *et al*. (2014) ^15^ where it is recommended that data is collected on a minimum of 60 patients per arm to estimate an ‘event’ rate in a single treatment arm. We planned to calculate a pooled estimate of overall recruitment rate and overall biomarker prevalence rate with a planned recruitment target of 70 patients in total to allow for dropout.

The target was to recruit 70 patients from the three centres: Newcastle, Glasgow and Cardiff. Participants were randomised using a method of random permuted blocks of concealed variable block size and stratified by site in the ratio 1:1 to receive personalised standard treatment (intervention) or standard care (control). In the personalised standard treatment group, participants’ treatment was guided by the results of the AR-V7 biomarker test. Participants randomised to the control arm received standard care without biomarker guided treatment. Details of the protocol for the blood sampling, processing and analyses are previously published ^13^. In brief, 2 x 10ml blood samples using ACD-A Blood Collection Tubes were collected at baseline, 12 and 24 weeks. These samples were sent to Newcastle University on the day of collection using a courier service and shipped at a temperature below 10°C, i.e. on cool packs, but not frozen. These samples were used for CTC and cfDNA analysis and to provide the AR-V7 biomarker result using a validated commercially available kit - AdnaTest ProstateCancerPanel AR-V7 circulating tumour cell (CTC) quantitative RT-PCR (RT-qPCR) assay (Qiagen®) ^16^.

Outcome measures were defined as feasibility measures to inform the definitive RCT and clinical measures. Feasibility measures included recruitment rates, proportion of patients who were eligible, the proportion who agreed to be randomised, the baseline prevalence of AR-V7 expression, how assessable blood samples were for the biomarker and timeline involved in processing the samples and whether patients were compliant with the recommended treatment and completing study measures. Clinical measures included time to prostate specific antigen (PSA) progression, clinical progression, cancer specific and overall survival. Clinical progression could be determined as a result of progressive symptomology or radiologically. As VARIANT was a pragmatic trial the latter was as determined by local radiology or multidisciplinary team.

Further to this quality of life (QOL) was assessed at baseline, 12 weeks and 24 weeks using the validated EORTC quality of life cancer questionnaires (QLQ-C30) with additional prostate cancer specific module (QLQ-PR25) ( https://qol.eortc.org/). Additionally, a short non-validated ‘Use of Health Services Questionnaire’, consisting of ten questions assessing how patients utilised health resources during the trial was completed once at the end of the trial, to aid in future health economic evaluation, this is available as extended data ^17^.

Further information about the methods including detailed eligibility criteria and outcomes is available in the earlier published protocol ^13^.

## Results

### Recruitment, eligibility, randomisation and baseline demographics

**Figure 1.**
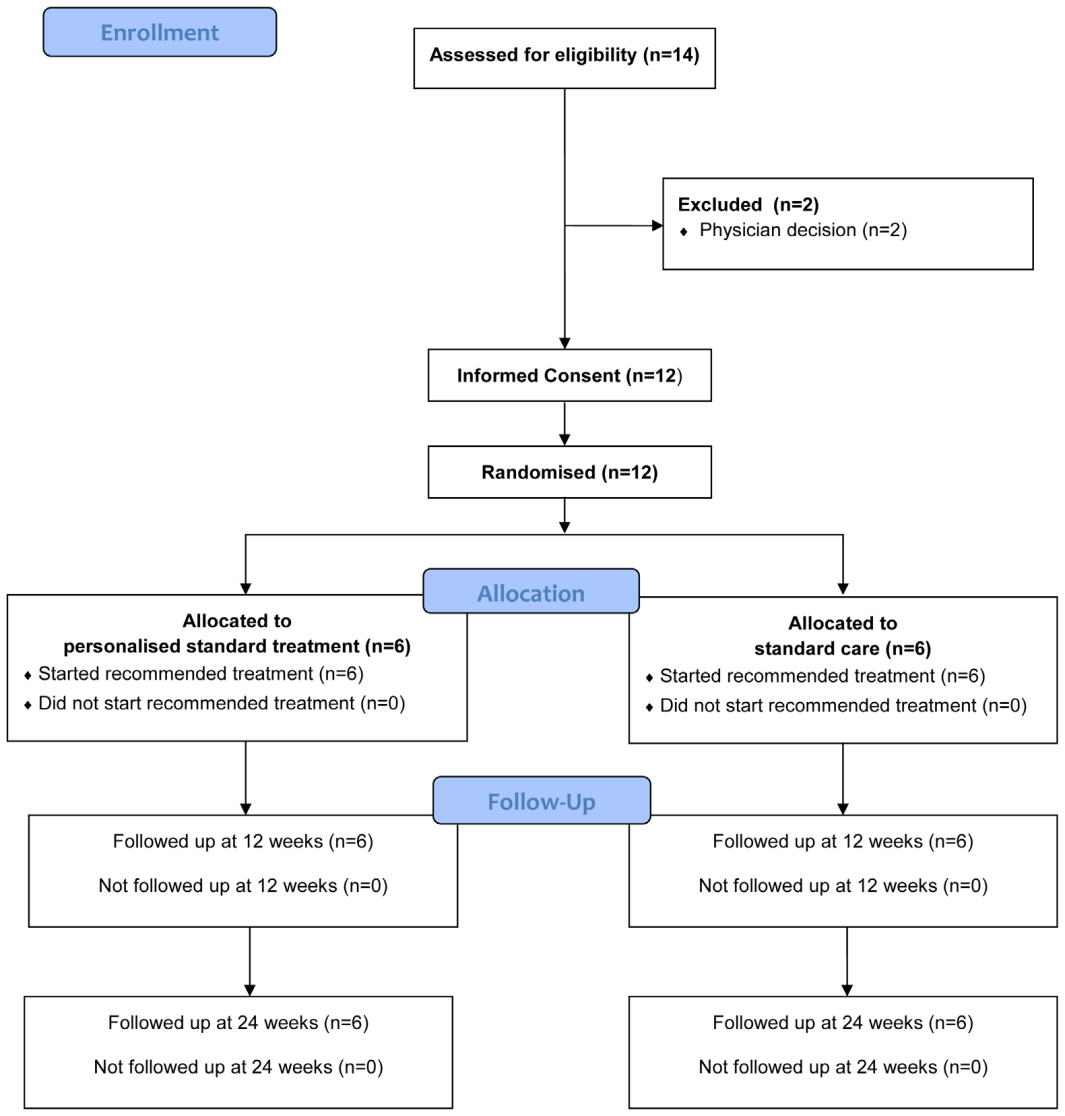
CONSORT flow diagram.

Participant flow is summarised in the Consolidated Standards of Reporting Trials (CONSORT) diagram in Figure 1, additionally the CONSORT checklist is available as extended data ^17, 18^. Of the 14 patients who were assessed against eligibility criteria, two patients were excluded as they were deemed too unfit to participate. Of the remaining 12 patients, all 12 agreed to be randomised with six patients randomised into each arm, four of these patients were randomised to have validation blood samples sent to the Cardiff labs. No participants withdrew or were lost to follow up over the course of the study. Baseline demographics are provided in Table 1.

**Table 1.** Clinical demographics and medical history of participants at screening.

		Personalised Treatment (n = 6)	Standard Care (n = 6)
*Disease characteristics (at initial diagnosis)*			
**PSA**	median (range) **(ng/ml)**	*116.7 (14.1–436)*	*16.25 (1.7–991)*
**Gleason score**	**6 | 7 | 8 | 9 | 10**	*0 | 1 (17%) | 2 (33%) | 3 (50%) | 0*	*1 (17%) | 0 | 2 (33%) | 3 (50%) | 0*
**TNM ^a^** **Stage**	**T1 | T2 | T3 | T4**	*0* | *0* | *5 (83%)* | *1 (17%)*	*0* | *1(17%)* | *0* | *5 (83%)*
	**N0 | N1**	*3 (50%)* | *3 (50%)*	*4 (67%)* | *2 (33%)*
	**M0 | M1**	*1 (17%)* | *5 (83%)*	*4 (67%)* | *1 (33%)*
**ECOG ^b^** **PS ^c^**	**0 | 1**	*5 (83%)* | *1 (17%)*	*4 (67%)* | *2 (33%)*
*At entry to Variant*			
**Age**	median (range) **years**	*70.2 (62.0–76.9)*	*66.4 (55.4–74.2)*
**Time since diagnosis**	median (range) **years**	*1.4 (0.79–11.5)*	*3.1 (0.95–8.77)*
**Metastatic disease** **location**	**Bone** **Visceral** **Lymph Node**	*4 (67%)* *2 (33%)* *2 (33%)*	*4 (67%)* *0 (0%)* *2 (33%)*

*^a^Tumour, Nodes and Metastases, ^b^Eastern Cooperative Oncology Group ^c^performance status*

### AR-V7 analysis

All participants had blood samples taken and results emailed back to respective sites in a timeframe amenable to commence biomarker guided treatment. Median time between blood sample collection and result being received was 6.5 (3–7) days and nine (3–30) days between sample collection and treatment starting respectively. For the six participants in the personalised treatment arm, five participants were reported as AR-V7 negative and one participant was AR-V7 positive.

Following these results, issues were discovered with the AR-V7 assays used at the Newcastle Lab, as there was a failure of reproducibility with discrepancies found with those undergoing validation in the Cardiff lab - although some variation is to be expected since the number of circulating tumour cells can vary between vials of blood. In 9 out of 12 participants, an internal control for the AR-V7 assay (the inhibition control) failed. This meant that the team could not be confident of the reliability of the results for these participants. Further investigation and discussion with the provider suggested that this was an issue with the batch of assays being used. As VARIANT was a pragmatic trial, it was decided by the trial management group (TMG) that a second blood sample would not be sought from the participants. As a result, further investigations, including the impact of varying numbers of circulating tumour cells between vials of bloods were not completed.

Due to these issues and the low number of participants recruited, it was not possible to accurately calculate AR-V7 biomarker prevalence in this trial population. Issues faced with some of the assays withstanding, we were able to demonstrate the effective set up of a bespoke lab that allowed reporting of AR-V7 reads from blood samples in a timeline that could inform treatments for men with mCRPC.

### Treatment adherence and disease progression

Within the six participants allocated to the personalised treatment arm the five negative participants were started on next generation hormonal treatment (Enzalutamide or Abiraterone) and the one participant with a positive AR-V7 result started on chemotherapy in the form of Cabazitaxel.

Three patients had evidence of disease progression at 12 weeks, two in the personalised treatment arm and one in the standard care arm, all three had evidence of PSA and clinical progression. Two participants, one from each arm, changed therapy, in both cases from Enzalutamide to Cabazitaxel and in one case with the addition of Denosumab. This is summarised in Table 2. There were no deaths reported during the trial period.

**Table 2.** Summary of treatment received by participants.

		Personalised Treatment (n = 6)	Standard Care (n = 6)
AR-V7 analysis (baseline):			
Positive		*1 (17%)*	*NA*
Negative		*5 (83%)*	*NA*
Treatment recommendations: (based on AR-V7 status in the personalised treatment arm or per standard practice in the standard care arm)			
**Non-hormonal:**		***1 (17%)***	***1 (17%)***
Docetaxel chemotherapy		*0 (0%)*	*0 (0%)*
Cabazitaxel chemotherapy		*1 (17%)*	*1 (17%)*
Radium-223 therapy		*0 (0%)*	*0 (0%)*
**Next generation hormonal:**		***5 (83%)***	***5 (83%)***
Enzalutamide		*2 (33%)*	*2 (33%)*
Abiraterone		*3 (50%)*	*3 (50%)*
**Did the participant start recommended treatment?**	Yes No	*6 (100%)*	*6 (100%)*
		*0 (0%)*	*0 (0%)*
Treatment received			
**Did the participant change anti-cancer therapy over the** **course of the trial?**		*1 (17%)* *Enzalutamide* *to Cabazitaxel* *+ Denosumab*	*1 (17%)* *Enzalutamide to* *Cabazitaxel*

### Quality of life

The majority of participants completed quality of life questionnaires (EORTC QLQ-C30 & PR25) at baseline (n=11) and again (n=10) at 24 weeks. Health Service questionnaires were completed by the nine participants recruited in Newcastle but participants in Cardiff were erroneously not given the questionnaire to complete.

### Recruitment issues and trial end

**Figure 2.**
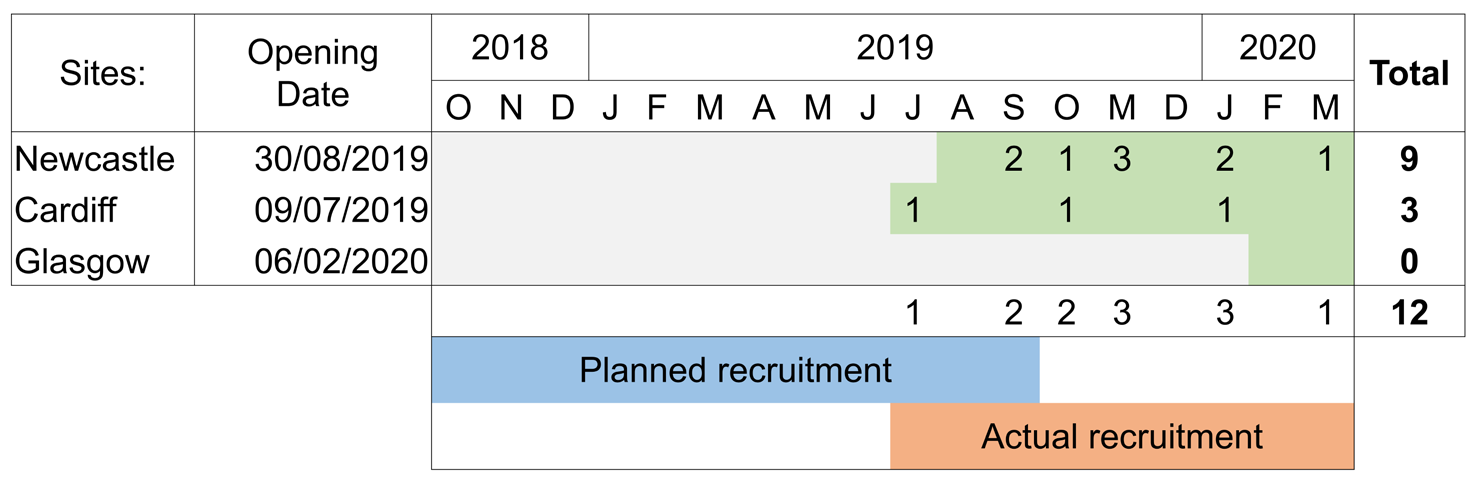
Recruitment timeline by month and site, whereby green shading indicates open for recruitment.

Due to delayed site opening, the recruitment period lasted less than nine-months rather than the full 12-month recruitment period that was planned. The opening of the three sites was projected to complete by the start of October 2018, however the first site started recruiting in July 2019 and all three sites were not fully operational until February 2020. This timeline and associated recruitment rates are shown in Figure 2. Recruitment rates were significantly lower than expected with an average of just over one participant recruited per month over the nine-month recruitment period, in contrast to a target of six participants per month. This was due to multiple reasons including slower timelines for sites opening to recruitment than originally planned and changes in clinical management pathway. The clinical management of metastatic prostate cancer evolved during study set up and early recruitment. This included the up-front use of docetaxel, though off license at the time, recommended in new NICE guidance published in May 2019 ^19^. As a result the management of men with metastatic prostate cancer now involves treatment with either novel hormonal therapies and/or chemotherapy prior to the onset of castration resistance and the conventional mCRPC stage we were examining is now uncommonly seen.

In addition to the above, further delays were caused by regulatory approval, this was secondary to the decision by the European Medicines Agency to restrict the use of radium-223, one of the non-hormonal treatment options ^20^. The AR-V7 assay being used in the study also changed (non-CE marked). Both of these changes required review of regulatory requirements and a delay in submission of ethics approval, pushing back all subsequent milestones. 

The trial management team explored alternative measures to increase the rates of recruitment including the addition of extra sites. Southampton Hospital and University Hospital Bristol had been approached to take part however, the changing clinical management pathways and other competing studies meant that the new sites would face similar, if not the same, issues faced at the initial three recruiting sites. Nevertheless, we were able to demonstrate that some men with advanced cancer were willing to randomise to a study of biomarker-directed therapy

Following a Trial Oversight Committee meeting, recruitment to the trial was halted on 18 ^th^ March 2020 at all sites. As a result of the COVID-19 pandemic (also March 2020), the central Newcastle University laboratories were forced to close. No blood samples were collected from participants for their follow up visits; however, participants were asked to complete the questionnaires remotely (sent to them via post or completed with a research nurse over the phone).

## Discussion

VARIANT successfully recruited 12 men to be randomised for biomarker guided treatment for prostate cancer and followed them up for 24 months. Though it failed to recruit as planned, this doesn’t appear to be due to a lack of willingness by clinicians or patients, with only two screen failures reported within the study, both secondary to patient fitness. The aforementioned delays impacted negatively with our ability to keep up with the rapidly changing field of prostate cancer management; we were aware of the potential for changes in treatment practice but had expected to fully recruit before these were realised. In the last few years there have been major changes in clinical practice with results from multiple clinical trials. For example, STAMPEDE, GETUG and CHAARTED compared ADT in mHSPC to ADT combined with docetaxel and found the addition of docetaxel up front led to an overall survival advantage ^21– 23^. In time this led to newly diagnosed mHSPC patients being treated with ADT + Docetaxel if fit enough. Multiple trials investigating the role of ADT and ARTA (with or without chemotherapy) have now also published their results leading to further direct changes in both the management of patients with mHSPC and an indirect shift in the care of these men when they develop mCRPC ^21, 24– 27^. These changed the management pathway of the patient cohort selected for this trial, as treatment at the time of development of castrate resistance is dependent on the prior treatment given ^28^. Though this issue is not intrinsic to VARIANT, it did influence our ability to recruit participants, and changed the validity of the underlying hypothesis of the trial. Another factor was the assay reproducibility failures that hindered our initial AR-V7 status reporting. This was quickly recognised due to our planned cross-site validation of blood samples and the cause identified. We would highlight the importance of cross site validation for future biomarker trials along with ensuring adequate time and funding is allocated to testing assay reliability prior to commencement.

Clinical trials not meeting their objectives is by no means uncommon, with results often going unpublished. One study reviewing trials of 640 novel therapeutics found that 344 did not continue in clinical development and of these only 40% had their results published in peer reviewed journals ^29^. Whilst the most common cause of difficulties experienced in those trials for novel therapeutics was inadequate efficacy, our experience with unsuccessful recruitment was found to be the most common identified within both urology and oncology trials ^30– 32^. Bandari *et al*. identified 1340 clinical trials in urology over a 10-year period of which 618 were unsuccessful, 41% of there were attributed to poor accrual, other causes included inadequate budget (9%), sponsor cancellation (7%) and poor interim results (7%). Within urology trials a significant association was found between unsuccessful trials and trials within oncology or andrology, device trials and trials funded by a combination of government and industry grants ^30^. Furthermore, a study in the UK across all specialities looking at trials funded by the MRC and HTA between 1994–2002 found that only 31% of studies recruited 100% of their original target and 45% achieved <80% of their original target, with 30% of trials reducing their recruitment targets and 54% requesting a trial extension ^33^.

Within VARIANT recruitment was well below the estimated level, with one site not recruiting a single patient. Successful recruitment has previously found to be associated with trial sites with prior track record of successful trials and also trial staff enthusiasm ^34^. Levitt *et al*. looked at recruitment levels across a number of sites in a large perinatal trial and identified factors associated with improved recruitment. They found that clearly defined recruitment systems, staff engagement, having a dedicated and experienced trial coordinator and a shorter time taken from ethics approval to first recruit were all associated with above average recruitment ^35^. They concluded that it may be better to focus resources on fewer sites with adequate resources and engaged staff ^35^. A formal process evaluation, such as the Quintet (Qualitative Research Integrated within Trials) recruitment intervention (QRI), could have helped identify barriers to recruitment, but was not part of the funded protocol. For feasibility studies, where there are predicted concerns about recruitment, a QRI to explore barriers and develop plans to optimise recruitment could be useful ^36^

Another method to try and improve trial success is the use of adaptive trial design whereby outcomes are assessed at pre-defined points and can be modified based on pre-specified rules. As a result, use of resources can be more efficient and potentially fewer patients may be required ^37, 38^. One such example of this in urology is the STAMPEDE trial, briefly mentioned earlier, which examines systematic therapy in advancing or metastatic prostate cancer ^21, 39^. Another technique being assessed to improve trial design and increase success rates is artificial intelligence. Proposed applications include machine learning techniques used to enhance patient recruitment through automatic eligibility assessment and trial recommendation ^40^.

AR-V7 remains clinically relevant with a recent systematic reviews finding a positive AR-V7 status to be associated with a reduced overall survival (OS) in comparison to AR-V7 negative patients ^41– 43^. This was the case for both ARTA treatment and chemotherapy, although to a lesser extent in the latter. Where treatment response was compared chemotherapy was associated with a superior survival in AR-V7 positive patients than those treated with ARTA, this difference was not observed in those who were AR-V7 negative ^42, 43^. Whilst some studies have continued to examine its use as a biomarker and further develop assays other studies are exploring the means to directly target the AR-V7 variants to overcome hormone resistance ^44– 47^.

## Conclusions

We present the results of the VARIANT clinical trial looking at the AR-V7 biomarker to guide treatment for patients with mCRPC. We can conclude that some men with prostate cancer are willing to take part in trials utilising biomarker guided treatment. However, due to issues with recruitment secondary to unforeseen delays and change within the management of prostate cancer the trial did not complete as planned. The lessons learned from this pilot trial are applicable to other research particularly in relation to fields where there is a rapid advance in knowledge.

## Abbreviations

CRPC: castrate resistant prostate cancer

AR-V7: androgen receptor splice variant 7

NHS: National Health Service

ADT: androgen deprivation therapy

mHSPC: metastatic hormone sensitive prostate cancer

mCRPC: metastatic castrate resistant prostate cancer

ARTA: androgen receptor targeted agents

CTCs: circulating tumour cells

RCT: randomised control trial

PSA: prostate specific antigen

QOL: quality of life

CONSORT: Consolidated Standards of Reporting Trials

ECOG: Eastern Cooperative Oncology Group

PS: Performance Status

TMG: trial management group

## Consent

Written informed consent for publication of the patients’ details was obtained from the patients.

## Data Availability

Underlying data from this study are available on request from the corresponding author, Rakesh Heer ( rakesh.heer@newcastle.ac.uk). The data is not available publicly due to confidentiality restrictions. Access to de-identified data collected during the trial, may be granted to researchers who submit a methodologically sound proposal. To gain access, data requestors will need to complete forms required as part of the application process. Zenodo: Extended data for ‘Using the AR-V7 biomarker to determine treatment in metastatic castrate resistant prostate cancer, a feasibility randomised control trial, conclusions from the VARIANT trial’. https://doi.org/10.5281/zenodo.6874339 ^17^ Zenodo: CONSORT checklist for ‘Using the AR-V7 biomarker to determine treatment in metastatic castrate resistant prostate cancer, a feasibility randomised control trial, conclusions from the VARIANT trial’. https://doi.org/10.5281/zenodo.6874339 ^17^ Data are available under the terms of the Creative Commons Attribution 4.0 International license (CC-BY 4.0)

## References

[1] Dyba T Randi G Bray F : The European cancer burden in 2020: Incidence and mortality estimates for 40 countries and 25 major cancers. *Eur J Cancer.* 2021;157:308–47. 10.1016/j.ejca.2021.07.039 34560371 PMC8568058

[2] Damodaran S Kyriakopoulos CE Jarrard DF : Newly Diagnosed Metastatic Prostate Cancer: Has the Paradigm Changed? *Urol Clin North Am.* 2017;44(4):611–621. 10.1016/j.ucl.2017.07.008 29107277 PMC6402766

[3] Crowley F Sterpi M Buckley C : A Review of the Pathophysiological Mechanisms Underlying Castration-resistant Prostate Cancer. *Res Rep Urol.* 2021;13:457–72. 10.2147/RRU.S264722 34235102 PMC8256377

[4] Dong L Zieren RC Xue W : Metastatic prostate cancer remains incurable, why? *Asian J Urol.* 2019;6(1):26–41. 10.1016/j.ajur.2018.11.005 30775246 PMC6363601

[5] Heidenreich A Bastian PJ Bellmunt J : EAU guidelines on prostate cancer. Part II: Treatment of advanced, relapsing, and castration-resistant prostate cancer. *Eur Urol.* 2014;65(2):467–79. 10.1016/j.eururo.2013.11.002 24321502

[6] Dehm SM Schmidt LJ Heemers HV : Splicing of a novel *androgen receptor* exon generates a constitutively active androgen receptor that mediates prostate cancer therapy resistance. *Cancer Res.* 2008;68(13):5469–77. 10.1158/0008-5472.CAN-08-0594 18593950 PMC2663383

[7] Nakazawa M Antonarakis ES Luo J : Androgen receptor splice variants in the era of enzalutamide and abiraterone. *Horm Cancer.* 2014;5(5):265–73. 10.1007/s12672-014-0190-1 25048254 PMC4167475

[8] Li Y Chan SC Brand LJ : Androgen receptor splice variants mediate enzalutamide resistance in castration-resistant prostate cancer cell lines. *Cancer Res.* 2013;73(2):483–9. 10.1158/0008-5472.CAN-12-3630 23117885 PMC3549016

[9] Sun S Sprenger CC Vessella RL : Castration resistance in human prostate cancer is conferred by a frequently occurring androgen receptor splice variant. *J Clin Invest.* 2010;120(8):2715–30. 10.1172/JCI41824 20644256 PMC2912187

[10] Hu R Dunn TA Wei S : Ligand-independent androgen receptor variants derived from splicing of cryptic exons signify hormone-refractory prostate cancer. *Cancer Res.* 2009;69(1):16–22. 10.1158/0008-5472.CAN-08-2764 19117982 PMC2614301

[11] Mostaghel EA Marck BT Plymate SR : Resistance to CYP17A1 inhibition with abiraterone in castration-resistant prostate cancer: induction of steroidogenesis and androgen receptor splice variants. *Clin Cancer Res.* 2011;17(18):5913–25. 10.1158/1078-0432.CCR-11-0728 21807635 PMC3184252

[12] Nadiminty N Tummala R Liu C : NF-κB2/p52 induces resistance to enzalutamide in prostate cancer: role of androgen receptor and its variants. *Mol Cancer Ther.* 2013;12(8):1629–37. 10.1158/1535-7163.MCT-13-0027 23699654 PMC3941973

[13] Clark E Morton M Sharma S : Prostate cancer androgen receptor splice variant 7 biomarker study - a multicentre randomised feasibility trial of biomarker-guided personalised treatment in patients with advanced prostate cancer (the VARIANT trial) study protocol. *BMJ Open.* 2019;9(12):e034708. 31857319 10.1136/bmjopen-2019-034708 PMC6937062

[14] Eldridge SM Lancaster GA Campbell MJ : Defining Feasibility and Pilot Studies in Preparation for Randomised Controlled Trials: Development of a Conceptual Framework. *PLoS One.* 2016;11(3):e0150205. 10.1371/journal.pone.0150205 26978655 PMC4792418

[15] Teare MD Dimairo M Shephard N : Sample size requirements to estimate key design parameters from external pilot randomised controlled trials: a simulation study. *Trials.* 2014;15:264. 10.1186/1745-6215-15-264 24993581 PMC4227298

[16] Antonarakis ES Lu C Luber B : Clinical Significance of Androgen Receptor Splice Variant-7 mRNA Detection in Circulating Tumor Cells of Men With Metastatic Castration-Resistant Prostate Cancer Treated With First- and Second-Line Abiraterone and Enzalutamide. *J Clin Oncol.* 2017;35(19):2149–56. 10.1200/JCO.2016.70.1961 28384066 PMC5493048

[17] Gravestock P Clark E Morton M : Extended data for ’Using the AR-V7 biomarker to determine treatment in metastatic castrate resistant prostate cancer, a feasibility randomised control trial, conclusions from the VARIANT’.[Dataset].2022. 10.5281/zenodo.6874339 PMC7614403 37035713

[18] Schulz KF Altman DG Moher D : CONSORT 2010 Statement: updated guidelines for reporting parallel group randomised trials. *BMC Med.* 2010;8:18. 10.1186/1741-7015-8-18 20334633 PMC2860339

[19] Overview: Prostate cancer: Diagnosis and management: Guidance. NICE, (n.d.). Retrieved December 14, 2022. Reference Source

[20] O'Sullivan JM Heinrich D James ND : The Case Against the European Medicines Agency's Change to the Label for Radium-223 for the Treatment of Metastatic Castration-resistant Prostate Cancer. *Eur Urol.* 2019;75(3):e51–e52. 10.1016/j.eururo.2018.11.003 30454914

[21] James ND Sydes MR Clarke NW : Addition of docetaxel, zoledronic acid, or both to first-line long-term hormone therapy in prostate cancer (STAMPEDE): survival results from an adaptive, multiarm, multistage, platform randomised controlled trial. *Lancet.* 2016;387(10024):1163–77. 10.1016/S0140-6736(15)01037-5 26719232 PMC4800035

[22] Fizazi K Faivre L Lesaunier F : Androgen deprivation therapy plus docetaxel and estramustine versus androgen deprivation therapy alone for high-risk localised prostate cancer (GETUG 12): a phase 3 randomised controlled trial. *Lancet Oncol.* 2015;16(7):787–94. 10.1016/S1470-2045(15)00011-X 26028518

[23] Sweeney CJ Chen YH Carducci M : Chemohormonal Therapy in Metastatic Hormone-Sensitive Prostate Cancer. *N Engl J Med.* 2015;373(8):737–46. 10.1056/NEJMoa1503747 26244877 PMC4562797

[24] Fizazi K Tran N Fein L : Abiraterone plus Prednisone in Metastatic, Castration-Sensitive Prostate Cancer. *N Engl J Med.* 2017;377(4):352–60. 10.1056/NEJMoa1704174 28578607

[25] Chi KN Agarwal N Bjartell A : Apalutamide for Metastatic, Castration-Sensitive Prostate Cancer. *N Engl J Med.* 2019;381(1):13–24. 10.1056/NEJMoa1903307 31150574

[26] Davis ID Martin AJ Stockler MR : Enzalutamide with Standard First-Line Therapy in Metastatic Prostate Cancer. *N Engl J Med.* 2019;381(2):121–31. 10.1056/NEJMoa1903835 31157964

[27] Armstrong AJ Szmulewitz RZ Petrylak DP : ARCHES: A Randomized, Phase III Study of Androgen Deprivation Therapy With Enzalutamide or Placebo in Men With Metastatic Hormone-Sensitive Prostate Cancer. *J Clin Oncol.* 2019;37(32):2974–86. 10.1200/JCO.19.00799 31329516 PMC6839905

[28] Cornford P van den Bergh RCN Briers E : EAU-EANM-ESTRO-ESUR-SIOG Guidelines on Prostate Cancer. Part II-2020 Update: Treatment of Relapsing and Metastatic Prostate Cancer. *Eur Urol.* 2021;79(2):263–282. 10.1016/j.eururo.2020.09.046 33039206

[29] Hwang TJ Carpenter D Lauffenburger JC : Failure of Investigational Drugs in Late-Stage Clinical Development and Publication of Trial Results. *JAMA Intern Med.* 2016;176(12):1826–33. 10.1001/jamainternmed.2016.6008 27723879

[30] Bandari J Theisen KM Maganty A : Clinical Trials in Urology: Predictors of Successes and Failures. *J Urol.* 2020;204(4):805–10. 10.1097/JU.0000000000001072 32267191

[31] Stensland KD McBride RB Latif A : Adult cancer clinical trials that fail to complete: an epidemic? *J Natl Cancer Inst.* 2014;106(9):dju229. 10.1093/jnci/dju229 25190726

[32] Nguyen TK Nguyen EK Warner A : Failed Randomized Clinical Trials in Radiation Oncology: What Can We Learn? *Int J Radiat Oncol Biol Phys.* 2018;101(5):1018–24. 10.1016/j.ijrobp.2018.04.030 29859791

[33] McDonald AM Knight RC Campbell MK : What influences recruitment to randomised controlled trials? A review of trials funded by two UK funding agencies. *Trials.* 2006;7:9. 10.1186/1745-6215-7-9 16603070 PMC1475627

[34] Fogel DB : Factors associated with clinical trials that fail and opportunities for improving the likelihood of success: A review. *Contemp Clin Trials Commun.* 2018;11:156–64. 10.1016/j.conctc.2018.08.001 30112460 PMC6092479

[35] Levett KM Roberts CL Simpson JM : Site-specific predictors of successful recruitment to a perinatal clinical trial. *Clin Trials.* 2014;11(5):584–9. 10.1177/1740774514543539 25055812

[36] Rooshenas L Scott LJ Blazeby JM : The QuinteT Recruitment Intervention supported five randomized trials to recruit to target: a mixed-methods evaluation. *J Clin Epidemiol.* 2019;106:108–20. 10.1016/j.jclinepi.2018.10.004 30339938 PMC6355457

[37] Pallmann P Bedding AW Choodari-Oskooei B : Adaptive designs in clinical trials: why use them, and how to run and report them. *BMC Med.* 2018;16(1):29. 10.1186/s12916-018-1017-7 29490655 PMC5830330

[38] Bothwell LE Avorn J Khan NF : Adaptive design clinical trials: a review of the literature and ClinicalTrials.gov. *BMJ Open.* 2018;8(2):e018320. 10.1136/bmjopen-2017-018320 29440155 PMC5829673

[39] Hague D Townsend S Masters L : Changing platforms without stopping the train: experiences of data management and data management systems when adapting platform protocols by adding and closing comparisons. *Trials.* 2019;20(1):294. 10.1186/s13063-019-3322-7 31138292 PMC6540437

[40] Harrer S Shah P Antony B : Artificial Intelligence for Clinical Trial Design. *Trends Pharmacol Sci.* 2019;40(8):577–91. 10.1016/j.tips.2019.05.005 31326235

[41] Liu RJ Hu Q Li SY : The Role of Androgen Receptor Splicing Variant 7 in Predicting the Prognosis of Metastatic Castration-Resistant Prostate Cancer: Systematic Review and Meta-Analysis. *Technol Cancer Res Treat.* 2021;20:15330338211035260. 10.1177/15330338211035260 34313171 PMC8320551

[42] Khan T Becker TM Scott KF : Prognostic and Predictive Value of Liquid Biopsy-Derived Androgen Receptor Variant 7 (AR-V7) in Prostate Cancer: A Systematic Review and Meta-Analysis. *Front Oncol.* 2022;12:868031. 10.3389/fonc.2022.868031 35372002 PMC8971301

[43] Wang Z Shen H Ma N : The Prognostic Value of Androgen Receptor Splice Variant 7 in Castration-Resistant Prostate Cancer Treated With Novel Hormonal Therapy or Chemotherapy: A Systematic Review and Meta-analysis. *Front Oncol.* 2020;10:572590. 10.3389/fonc.2020.572590 33425724 PMC7793884

[44] Moon SJ Jeong BC Kim HJ : Bruceantin targets HSP90 to overcome resistance to hormone therapy in castration-resistant prostate cancer. *Theranostics.* 2021;11(2):958–73. 10.7150/thno.51478 33391515 PMC7738850

[45] Shenderov E Boudadi K Fu W : Nivolumab plus ipilimumab, with or without enzalutamide, in AR-V7-expressing metastatic castration-resistant prostate cancer: A phase-2 nonrandomized clinical trial. *Prostate.* 2021;81(6):326–38. 10.1002/pros.24110 33636027 PMC8018565

[46] del Re M Conteduca V Crucitta S : Androgen receptor gain in circulating free DNA and splicing variant 7 in exosomes predict clinical outcome in CRPC patients treated with abiraterone and enzalutamide. *Prostate Cancer Prostatic Dis.* 2021;24(2):524–31. 10.1038/s41391-020-00309-w 33500577 PMC8134038

[47] Lu D Krupa R Harvey M : Development of an immunofluorescent AR-V7 circulating tumor cell assay - A blood-based test for men with metastatic prostate cancer. *J Circ Biomark.* 2020;9:13–9. 10.33393/jcb.2020.2163 33717359 PMC7951184

