# Supplementary material for: Using the AR-V7 biomarker to determine treatment in metastatic castrate resistant prostate cancer, a feasibility randomised control trial, conclusions from the VARIANT trial
Source: NIHR Open Res. 2023 Jan 10;2:49. Originally published 2022 Sep 2. [Version 2] doi: 10.3310/nihropenres.13284.2 (PMC7614403; doi:10.3310/nihropenres.13284.2)
Supplement: Peer review history

# Reviewer report: Reviewer response for version 2

**DOI:** 10.3310/nihropenres.14489.r28945

**Published:** 2023-02-07

**Authors:** Jonathan J. Aning

**Affiliations:** 1 University of Bristol, Bristol, UK; 2 Bristol Urological Institute, North Bristol NHS Trust, Bristol, UK

No further comments to make.

Is the work clearly and accurately presented and does it cite the current literature?

Yes

If applicable, is the statistical analysis and its interpretation appropriate?

Not applicable

Are all the source data underlying the results available to ensure full reproducibility?

No source data required

Is the study design appropriate and is the work technically sound?

Yes

Are the conclusions drawn adequately supported by the results?

Yes

Are sufficient details of methods and analysis provided to allow replication by others?

Yes

Reviewer Expertise:

NA

I confirm that I have read this submission and believe that I have an appropriate level of expertise to confirm that it is of an acceptable scientific standard.

# Reviewer report: Reviewer response for version 2

**DOI:** 10.3310/nihropenres.14489.r28946

**Published:** 2023-01-25

**Authors:** Therese M. Becker

**Affiliations:** 1 Centre for Circulating Tumour Cell Diagnostics and Research, Ingham Institute for Applied Medical Research, Liverpool, NSW, Australia

Approved, the authors have addressed my issues.

Is the work clearly and accurately presented and does it cite the current literature?

Partly

If applicable, is the statistical analysis and its interpretation appropriate?

Not applicable

Are all the source data underlying the results available to ensure full reproducibility?

No

Is the study design appropriate and is the work technically sound?

Partly

Are the conclusions drawn adequately supported by the results?

Partly

Are sufficient details of methods and analysis provided to allow replication by others?

No

Reviewer Expertise:

AR-V7 testing in liquid biopsies.

I confirm that I have read this submission and believe that I have an appropriate level of expertise to confirm that it is of an acceptable scientific standard.

# Reviewer report: Reviewer response for version 1

**DOI:** 10.3310/nihropenres.14404.r28873

**Published:** 2022-11-29

**Authors:** Jonathan J. Aning

**Affiliations:** 1 University of Bristol, Bristol, UK; 2 Bristol Urological Institute, North Bristol NHS Trust, Bristol, UK

The present manuscript describes the outcomes and learning from the VARIANT trial. This feasibility study was designed to find out whether men could be randomised to either receiving a biomarker assay followed by a personalised treatment guided by the assay result or routine standard of care treatment. The study did not meet its planned recruitment target and the reasons for this are outlined in the manuscript. Although the study participant numbers were low, the authors showed that patient retention and compliance with study procedures and protocol for those men recruited was good.

 Firstly the authors are to be commended for writing up the VARIANT trial. Their experience and the shared study findings represent important learning for future prostate cancer studies (and biomarker studies in general). Biomarker studies will invariably continue to form part of the future of researching personalised treatment strategies.

**Major comments**

- This study highlights the need to have rigorously evaluated the novel biomarker assay prior to commencing the study. In the future similar studies should consider incorporating time and funding for ensuring test reliability in the run in period to starting the study. Could the authors make a definitive comment on this within the discussion section of their manuscript and define in their opinion the optimum validation method?

I have no other major comments regarding the content of the manuscript but I have a number of minor comments that I feel that the authors should address to ensure that readers have both the context and information to fully understand the learning from this study.

**Minor comments**

*In the plain English summary:*

- The timeline of the study should be clearly defined and included to facilitate clear lay reader understanding, i.e.. 'The Variant trial was planned in...', 'The study recruitment commenced in...', 'The study closed in...'.

*In the Methodology:*

- Could the authors clarify if all patients included had to have a *de-novo* diagnosis of metastatic prostate cancer or were men who had had previous prostate cancer treatments who then developed metastatic prostate cancer also included?

*In the results section:*

- In the recruitment, eligibility, randomisation and baseline demographic section – please could you include the dates the trial opened for recruitment as this is critical for context of understanding why the recruitment of participants was so low.
- In the treatment adherence and disease progression section – please define what classed as ‘disease progression’ was it according to RECIST criteria?
- In the recruitment issues and trial end section – please define and reference the date when the clinical pathway changed – (e.g., NICE guidance for docetaxel chemotherapy / ARTA).

*In the discussion section:*

- Consider starting the discussion section with the achievements of the study – i.e. patients were recruited and retained.
- In the third paragraph of the Discussion – Comment on whether either QuinteT Recruitment strategies or enhanced PPI would have improved recruitment

Is the work clearly and accurately presented and does it cite the current literature?

Yes

If applicable, is the statistical analysis and its interpretation appropriate?

Not applicable

Are all the source data underlying the results available to ensure full reproducibility?

No source data required

Is the study design appropriate and is the work technically sound?

Yes

Are the conclusions drawn adequately supported by the results?

Yes

Are sufficient details of methods and analysis provided to allow replication by others?

Yes

Reviewer Expertise:

NA

I confirm that I have read this submission and believe that I have an appropriate level of expertise to confirm that it is of an acceptable scientific standard, however I have significant reservations, as outlined above.

# Response

**Published:** 2022-12-23

**Authors:** Paul Gravestock

**Affiliations:** Newcastle Upon Tyne Hospitals NHS Trust, UK

Thank you for your comments. We have added additional narrative around the issues faced with the biomarker assay to make things clearer within the result section (page 11). Additionally we have added a comment about validation within the first paragraph in the discussion (page 13).

 In terms of the minor comments, we have added more detail about the dates both in the plain english summary (page 6) and results section of the text (page 12) as this was previously only contained within figure 2. With regards to eligibility it was not exclusively de novo disease, the full eligibility criteria is discussed in detail in the published protocol which is refenced (page 7/8). As VARIANT was a pragmatic trial the use of criteria such as RECIST to define radiological progression was not mandated and used radiological progression was defined as per the local radiology/MDT, this has now been stated within the text (page 8). The change in NICE guidance has been reflected in the text (page 13) and changes within the discussion made as kindly suggested with a brief narrative on Quintet (page 15)

# Reviewer report: Reviewer response for version 1

**DOI:** 10.3310/nihropenres.14404.r28838

**Published:** 2022-11-11

**Authors:** Therese M. Becker

**Affiliations:** 1 Centre for Circulating Tumour Cell Diagnostics and Research, Ingham Institute for Applied Medical Research, Liverpool, NSW, Australia

This is a short report from a small feasibility clinical trial to base therapy decisions for CRPC patients on liquid biopsy testing of AR-V7. The limited conclusion of the early abandoned trial is that in principle patients would be happy to have their treatment guided by liquid biopsy analysis. The other lesson may be that changes in clinical practice may change “standard of care” treatment within a trials period diluting usefulness of data.

 While the outcome is limited there are other issues.

 Major issue:

1. It is noted that the nature/type of the AR-V7 detection assay is not detailed in the Methods. This is even though “issues” with this assay are a leading section in the Results part of the manuscript. It is clearly important what type of AR-V7 assay was done and how exactly* the test was conducted if critical "issues" with the assay are considered important and unexpected AR-V7 detection ratios led in part to abandoning the trial. Between the lines one can read that AR-V7 detection was based on CTCs as it is stated (without reference by the way) “…since the number of circulating tumour cells can vary between vials of blood.”
2.  I would like to see some deeper exploration of why or if in fact they really had issues with AR-V7 testing. Maybe AR-V7 is less prevalent in their patient population? Do they have any longitudinal data for individual patients to present? How exactly did they define “failure of reproducibility”?

Minor issues:

1. While the originally planned schematic diagram of the trial is interesting, a more relevant one would depict a diagram of the actual patients enrolled.
2. “….a recent systematic review finding a positive AR-V7 status to be an associated with a reduced overall survival (OS) in comparison to AR-V7 negative patients, though this was not observed in those treated with taxane based chemotherapy38.” Interstingly, our more recent systematic review (Khan et al 2022 ^1^) focusing on AR-V7 detected by liquid biopsy found AR-V7 positivity also affected response to taxanes.

*How much blood was taken? What type of collection tubes? How much blood was used for each CTC enrichment? By what technology/method was AR-V7 presence in a CTC sample tested?

Is the work clearly and accurately presented and does it cite the current literature?

Partly

If applicable, is the statistical analysis and its interpretation appropriate?

Not applicable

Are all the source data underlying the results available to ensure full reproducibility?

No

Is the study design appropriate and is the work technically sound?

Partly

Are the conclusions drawn adequately supported by the results?

Partly

Are sufficient details of methods and analysis provided to allow replication by others?

No

Reviewer Expertise:

AR-V7 testing in liquid biopsies.

I confirm that I have read this submission and believe that I have an appropriate level of expertise to confirm that it is of an acceptable scientific standard, however I have significant reservations, as outlined above.

## References

[1] : Prognostic and Predictive Value of Liquid Biopsy-Derived Androgen Receptor Variant 7 (AR-V7) in Prostate Cancer: A Systematic Review and Meta-Analysis. *Frontiers in Oncology* .2022;12: 10.3389/fonc.2022.868031 10.3389/fonc.2022.868031 PMC897130135372002

# Response

**Published:** 2022-12-23

**Authors:** Paul Gravestock

**Affiliations:** Newcastle Upon Tyne Hospitals NHS Trust, UK

Thank you for your comments. The full methodology is published and referenced within the text. Though we appreciate that given the nature of the study it would be prudent to include more details about the AR-V7 assay and the issues faced as you have suggested. Additional texts within the methodology and discussion sections have been added to reflect this. (pages 8 and 11 respectively).

 In terms of the minor comments, Figure 1 does reflect the actual flow of patients, n=14 screened, n=12 randomised. Additionally we thank you for signposting the recent evidence and we have amended the final paragraph of the discussion to include this novel review (page 15).
